# Enhancing Equity in Access and Quality of Youth Out-of-School-Time Recreational Activities: Perspectives from Primary Caregivers and Parents in Under-resourced Urban Communities Using Semi-structured Interviews

**DOI:** 10.1007/s10995-025-04179-3

**Published:** 2025-09-24

**Authors:** Jaime La Charite, Mercedes Santoro, Cindy Flores, Alejandra Hurtado, Meachelle Lum, Yelba Castellon-Lopez, Rebecca Dudovitz

**Affiliations:** 1 Division of General Internal Medicine and Health Services Research, David Geffen School of Medicine at University of California, Los Angeles, 1100 Glendon Ave. Suite 900, Los Angeles, CA 90024 USA; 2 County of Los Angeles Department of Parks and Recreation, Los Angeles, West, 1000 S. Freemont Ave., Alhambra, CA 92803 USA; 3 Irvine School of Medicine, University of California, Irvine, 10001 Health Sciences Road, Irvine, CA 92697 USA; 4 David Geffen School of Medicine at University of California, Los Angeles, 10833 Le Conte, Los Angeles, CA 90095 USA; 5 Division of Population Sciences, Department of Biomedical Sciences, Cancer Research Center for Health Equity, Cedars Sinai Medical Center, 6500 Wilshire Blvd, Suite 1511, Los Angeles, CA 90048 USA; 6 Department of Pediatrics, University of California, Los Angeles, California, 10833 LeConte Ave., 12-358 CHS, Los Angeles, CA 90095 USA

**Keywords:** Extracurriculars, Disparities, Parents, Caregivers, Qualitative, Health

## Abstract

**Objectives:**

Out-of-school-time recreational activities are linked to numerous socioemotional, health, and academic benefits for children. Racial and income disparities in participation persist, yet there is a lack of qualitative studies eliciting the experiences and input of primary caregivers to improve equitable access to high-quality recreational activities in marginalized communities. This study explores caregiver perceptions of the factors influencing motivations to enroll their child in activities, barriers to participation, how caregivers define quality programming, and caregiver recommendations to improve activity access and quality within under-resourced communities.

**Methods:**

We recruited primary caregivers of children aged 6–17 from under-resourced communities in an urban county by purposive sampling through urban parks and recreation and community organizations. We conducted semi-structured interviews using descriptive methodology with content thematic analysis.

**Results:**

Thirty-four interviews (17 English, 17 Spanish) revealed three key themes: primary caregivers (1) were highly motivated, believing that activities were facilitators of lifelong healthy living and wellbeing for children, families, and communities, (2) identified ongoing participation barriers while recognizing opportunities to improve equitable access, (3) described high-quality activities as those promoting safety, inclusivity, and enjoyment. Parents highlighted strategies to promote equitable, high-quality programming, including broad outreach, easy enrollment with accessible activities, low financial barriers, structural investments, staff and volunteer training, and family engagement.

**Conclusions for Practice:**

Organizations offering youth out-of-school-time activities should consider caregiver practical suggestions to potentially improve the uptake and equity of these programs, with the ultimate goal of supporting the well-being and healthy development of all children.

## Significance

What is Known?

What this Study Adds?

Child participation in recreational activities is associated with numerous health benefits. However, racial and socioeconomic participation disparities persist. Prior caregiver surveys identified some access barriers (e.g., cost, transportation).

The study offers a thematic analysis of caregiver interviews from under-resourced, predominantly Black and Latinx communities. New underexplored motivating factors (child, family, and community wellbeing), barriers (lack of awareness, enrollment challenges), and caregiver perceptions of quality (safety, inclusivity, enjoyment) were identified. Caregivers suggested new outreach, enrollment, and cost reduction strategies and recommendations for structural investments, staff and volunteer training, and family engagement to optimize engagement and quality.

## Introduction

Out-of-school time activities (“activities”) are optional, adult-supervised enrichment opportunities outside school time, including sports, arts, academic clubs, community programs, and summer experiences (Naff et al., 2024). Participation correlates with better academics and physical and socioemotional health, with benefits potentially extending into adulthood (“Committee on Summertime,” 2019; Easterlin et al., 2019; Gleeson et al., 2011; La Charite et al., 2023). Mechanisms linking activities to health outcomes include supportive adult and peer relationships, reduced screen time, skill development, and regular exercise (Easterlin et al., 2019; Gleeson et al., 2011; Guilmette et al., 2019; Mahoney et al., 2002; Oberle et al., 2020). Youth of color, immigrant youth, and youth from lower-income households may gain more benefits from participation than their more advantaged peers (Heath et al., 2022; Phillips et al., 2021).

Activity participation disparities exist at the intersection of race, ethnicity, and class within under-resourced communities. Children who identify as Black and/or Latinx, along with children in low-income households, report lower participation and higher unmet demand; nationally, 63% of families who identify as Black in the lowest income bracket reported participation versus 88% in the highest bracket (Afterschool Alliance, 2021, 2022; La Charite et al., 2023). These disparities coincide with community factors. Racially segregated neighborhoods - stemming from discriminatory policies - and neighborhood income affect resource distribution and the quality of amenities (America & Becoming, 2001; McKenzie et al., 2014; Schwartz et al., 2022). Community amenity availability correlates with activity participation (Donnelly et al., 2019; McKenzie et al., 2014).

Most research on disparities relies on non-peer-reviewed national caregiver surveys, revealing access barriers like cost, inconvenient locations and hours, transportation issues, limited program space, and cultural isolation (Afterschool Alliance, 2021). These surveys did not identify a lack of perceived benefit or quality as limiting participation (Afterschool Alliance, 2022). Existing qualitative studies with caregivers focused on specific activities or parental involvement (Duerden et al., 2013; Fisher et al., 2022; Ju et al., 2020). No known qualitative studies explore caregiver perspectives on improving youth engagement in predominantly Black and Latinx, under-resourced communities. Qualitative interviews enable caregivers to detail their experiences with youth programming and suggest ways to create a more accessible, quality, and equitable activity system that may not have been captured in surveys (Smith et al., 1995).

We conducted semi-structured interviews with caregivers of children ages 6–17 in under-resourced urban areas with predominantly Latinx and Black families. Our goal was to elucidate caregiver insights for redesigning out-of-school time activities to optimize access, quality, and equity. To that end, we sought to describe primary caregiver (1) motivations to have their child participate in activities, (2) perceptions of participation barriers and recommendations to reduce barriers, and (3) perceptions of activity quality and recommendations to enhance quality. For aims one and two, we interviewed caregivers whose children were engaged and not engaged in activities to capture perspectives along varying degrees of engagement.

## Methods

### Research Team

#### Personal Characteristics

Four bilingual (English/Spanish) female research assistants (RAs) with Bachelor’s or Master of Public Health degrees conducted the interviews. They attended two two-hour trainings on qualitative research methods, interview techniques, and content analysis. The first author, bilingual and experienced in qualitative methods, reviewed the transcripts and provided feedback.

#### Relationship with Participants

Participants had no prior relationships with the study team. During recruitment and consent, RAs explained the study’s goal of improving recreational activities. Linguistic and cultural congruence existed between RAs and Latinx participants; however, none of the RAs identified as Black or African American.

### Study Design

#### Methodological Orientation and Theory

Our study was informed by Education Training Research (ETR)’s Health Equity Framework’s four domains (*Systems of Power*, *Relationships and Networks*, *Individual Factors*, and *Physiological Pathways*) (ETR, 2024). We conceptualized that policies, processes, and practices drive differential activity access (*Systems of Power*), influence family and peer relationships (*Relationships and Networks*), promote the development of health attitudes, skills, and behaviors (*Individual Factors*), and impact physical, cognitive, and psychological health (*Physiologic Pathways*). These concepts informed the development of our interview guide.

We conducted qualitative semi-structured interviews to identify caregivers’ perspectives about equity and quality. Semi-structured interviews are appropriate for narrating diverse caregiver perspectives (Kallio et al., 2016). We followed published steps to develop our interview guide: identifying prerequisites for semi-structured interviews, retrieving and using previous knowledge, formulating a preliminary guide, pilot testing, and presenting the guide (Kallio et al., 2016). Additional diversity and inclusion questions were added after the first two interviews. Our interview guide (Table 1) included questions to elucidate caregiver suggestions to achieve equity in activity access and quality (*Systems of Power*) and caregiver perceptions of how activity participation influences relationships, behaviors, and health (*Relationships*,* Individual Factors*,* Physiologic Pathways*).

**Table 1:**
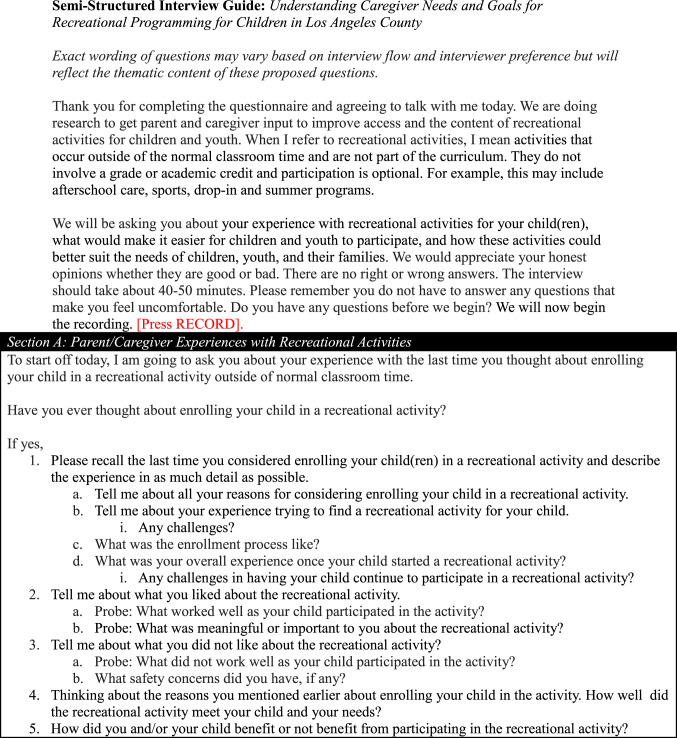

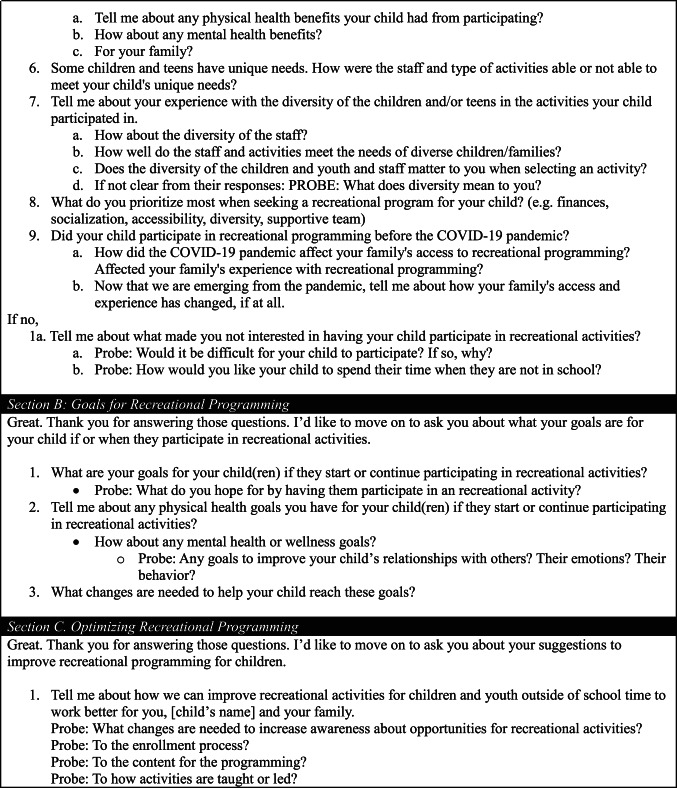

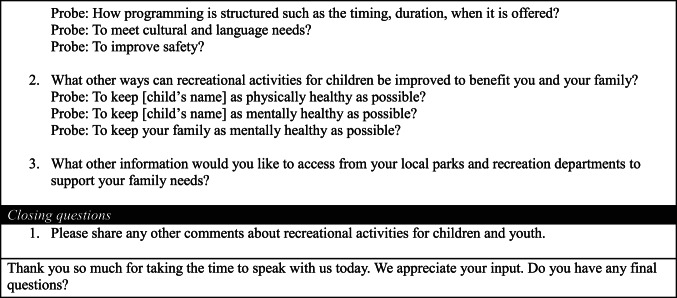
Interview guide

We used qualitative descriptive methodology and qualitative content analysis to describe the lived experiences of caregivers as they considered and navigated out-of-school-time programming to inform policy and program development rather than for theory development or meaning-making (Turale, 2020). Themes were data-driven rather than fit to a pre-existing theory.

#### Participant Selection

We prioritized caregivers from historically disinvested, predominantly Black and Latinx communities in East and South Los Angeles (LA) County. We sought to recruit families engaged and not engaged in activities to capture diverse perspectives. We used purposeful sampling recruiting across four categories: community park events in (1) East LA County and (2) South LA County, and community-based organizations engaging (3) Latinx residents in East LA County and (4) Black residents in South LA County. Community organizations received stipends to communicate study information to clients via email, social media, and community radio. RAs distributed recruitment flyers face-to-face during park events. Interested participants contacted the study team for eligibility screening.

Eligible participants were caregivers (18+) of children aged 6–17 living in East or South LA and accessible by email or phone. We interviewed 34 of 44 eligible caregivers (77%) until thematic saturation was reached (see *Data Analysis*).

### Setting

#### Community Context and Partnership

East and South LA County is home to 2 million residents – over 300,000 of whom are Black and nearly 1 million Latinx (Klein, 2001). These regions make up 23% of the county’s population and have high concentrations of residents identifying as Latinx or Black (South LA: 62% Latinx, 31% Black; East LA: 73% Latinx, 3% Black), foreign-born (36–40%), and report high poverty rates (16–30% vs. 15% countywide) (Los Angeles County, 2014; Ong and Firestine et al., 2008). We focused on areas that experience concentrated poverty, limited park access, and lower scores on the Equity Index, which measures poverty and social determinants of health (County of Los Angeles, California State Parks, 2024; Galperin, 2021; Ong et al., 2008). We suspect findings are transferable outside LA as other US urban environments similarly face disparities in green space access and have concentrated populations of Black and Latinx and low-income residents (Frey, 2021; Kephart, Lindsay, 2022).

In LA County, urban parks and recreation service providers, schools, and community organizations deliver activities. This study focused on general caregiver perceptions and recommendations rather than feedback directed at specific local organizations, increasing the transferability of the findings. Community-Partnered Participatory Research principles guided the study, engaging parks and recreation staff in formulating aims, developing an interview guide, recruiting participants, preparing the manuscript, and disseminating findings (Jones & Wells, 2007).

#### Data Collection and Setting

Participants completed a 15-minute web-based (or phone) pre-interview survey on demographics, health, and activity history. The survey was adapted from a prior study with Latinx community members with lower educational attainment (DeCamp et al., 2019). We included three questions from the National Survey of Children’s Health asking caregivers to rate their child’s physical and mental health and indicate if their child had a diagnosed medical condition (yes/no), since we were interested in including caregivers of children with and without health conditions (CAHMI, 2025).

A RA conducted a 45-minute semi-structured interview in English or Spanish with one participant by phone or Zoom (four requested Zoom). Interviews took place in a private location outside of the earshot of others. The interview guide (Table 1) covered motivations, experiences, barriers, facilitators, and recommendations for youth activities. Probes clarified and elaborated on responses. Interviews were audio-recorded, de-identified, and transcribed. Participants received a $50 gift card.

### Data Analysis

To describe the characteristics of our sample, we calculated frequencies and percentages for demographic items on the pre-interview survey stratified by language.

Transcripts were analyzed in five steps: (1) familiarization, (2) initial coding, (3) searching for themes, (4) refining themes, and (5) theme definition (Braun & Clarke, 2006). Transcripts were analyzed using Dedoose (v9.0.107) (Dedoose, 2024).

The coding team, consisting of four RAs supervised by the first author (JL), independently reviewed five transcripts, developed separate codebooks, and reconciled them through discussion. Two RAs then initially double-coded transcripts, and JL resolved discrepancies. This process continued until an interrater reliability score (kappa) of > 0.90 in Dedoose Training Center was achieved, after which transcripts were coded without a second reviewer. The four RAs and JL held weekly team meetings to address coding questions. Interviews and coding continued until thematic saturation, when no new information or codes emerged, was attained for both English and Spanish transcripts (Lincoln & Guba, 1985).

Following our inductive approach, we allowed themes to emerge from the data rather than fitting the data to a coding frame or pre-existing theory (Braun & Clarke, 2006). The Health Equity Framework informed the interview guide but did not drive data analysis. The coding team used Dedoose (Tong et al., 2007) visualization tools to review codes and organize themes (Braun & Clarke, 2006). They used group consensus to reconcile themes and reviewed them for internal consistency and distinctiveness (Braun & Clarke, 2006).

We used the Consolidated Criteria for Reporting Qualitative Research (COREQ) guidelines for reporting (Tong et al., 2007). IRB approval was obtained (IRB#23–000231).

## Results

We conducted 34 interviews (17 in English and 17 in Spanish). Sample demographics from the pre-interview survey are in Table 2. The analysis yielded a key theme for each study aim. Subthemes emerged to describe caregiver recommendations to improve motivation, access, and quality programming. Quotes are in-text and in Appendix Tables 5, 6 and 7. Tables 3 and 4 summarize caregiver recommendations for activity providers.

**Table 2:** Characteristics of interviewed participants stratified by preferred Language

	English (*N* = 16*)	Spanish (*N* = 17)
*Child Demographics*		
No. of children in household		
1 child	6 (35%)	4 (24%)
2 children	7 (41%)	8 (47%)
3 children	2 (12%)	4 (24%)
≥ 4 children	1 (6%)	1 (6%)
Missing	1 (6%)	0
Child Ages		
6–8 years	5 (29%)	2 (12%)
9–12 years	5 (29%)	7 (41%)
13–17 years	6 (35%)	8 (47%)
Missing	1 (6%)	0
Child Genders		
Female	3 (18%)	4 (24%)
Male	13 (76%)	13 (76%)
Nonbinary	0 (0%)	0 (0%)
Missing	1 (6%)	0
Child Physical Health		
Households with a child with fair or poor physical health	0 (0%)	2 (12%)
Child Mental Health		
Households with a child with fair or poor mental health	2 (12%)	2 (12%)
Child Diagnosed Condition		
Households with a child with a diagnosed health condition	7 (41%)	8 (47%)
Child Ever Participated in Out-of-School Time Activity		
Ever	16 (94%)	14 (82%)
Child Participated in Out-of-School Time Activity in Past 3 Months		
Ever	15 (88%)	8 (47%)
*Caregiver Demographics*		
Caregiver Gender		
Male	4 (24%)	2 (12%)
Female	12 (71%)	15 (88%)
Missing	1 (6%)	0
Caregiver Age		
18–29	0 (0.0%)	2 (12%)
30–39	8 (47%)	3 (18%)
40–49	7 (41%)	9 (53%)
50–59	1 (6%)	3 (18%)
Missing	1 (6%)	0
Country of Birth		
USA	14 (82%)	2 (12%)
Mexico	1 (6%)	11 (65%)
Central America	1 (6%)	4 (24%)
Missing	1 (6%)	0
Education		
< High School	0 (0%)	9 (53%)
High School	3 (18%)	6 (35%)
Some college	4 (24%)	0 (0%)
4-year university	8 (47%)	2 (12%)
Missing	1 (6%)	0
*Household Demographics*		
Annual Family Income		
< $20,000	2 (12%)	6 (35%)
$20,000–50,000	7 (41%)	10 (59%)
>$50,000	7 (41%)	1 (6%)
Missing	1 (6%)	0
Public Benefits		
Enrolled in Medicaid, WIC, or SNAP	12 (71%)	15 (88%)
Household Car		
No car in household	3 (18%)	2 (12%)

*One participant in the English-speaking category did not complete the pre-interview survey

* No.* Number

**Table 3:** Action statements to improve equitable access to out-of-school time programming based on caregiver interviews

	Action Statements for Community Organizations
Outreach	**Caregivers recommend comprehensive outreach strategies**. Caregivers suggested using email listservs, mailers, social media profiles, flyer distribution through schools and parks, and community networking via churches and local businesses. They also recommended a consistent physical and online location where all local organizations could post their activities. Enough information about the activity and its benefits should be provided to help caregivers motivate their children to attend.
Enrollment	**Caregivers recommend multiple easy-to-use ways to enroll in activities.** Caregivers recommend refining the enrollment process by providing in-person, telephone, and digital registration options, simplifying application questions, and establishing help lines (via internet portal, telephone number). It is important to caregivers that organizations are accessible in terms of language, literacy, and communication.
Accessibility	**Caregivers recommend investments to accommodate all interested families.** Caregivers recommend sufficient program capacity to allow all interested children to enroll in programming. Families also wish for flexibility in program timing to account for caregivers’ working schedules. Some caregivers also suggest offering programming multiple times a year and incorporating more summer activities. Lastly, families want to see more neighborhood public pools and larger parks, so families do not need to travel far to participate in programming.
Financial	**Caregivers recommend activity costs are low or free.** Families recommend keeping programming low-cost or free as finances can often be a barrier to registration and participation. What is considered “low-cost” ranged from $5-$50. Caregivers propose many suggestions, such as offering a waiver for caregivers who cannot afford associated costs, allowing payment plans, accepting payment on a lesson-by-lesson basis, developing a scholarship program, creating a system for caregivers to be able to borrow equipment and supplies, and making extra supplies/equipment are available. When an organization does offer financial supports, they should advertise openly so families are aware and can take advantage. To offset these costs to the organization, caregivers suggest organizations (1) find allies or sponsors who can provide uniforms, (2) host fundraisers, (3) partner with nonprofit organizations, (4) incorporate more volunteers, and (5) apply for grant funding. Beyond activities, caregivers also requested that these organizations offer referrals to other local community resources to address unmet basic needs.

**Table 4:** Action statements to improve the quality of out-of-school time programming based on caregiver interviews

	Action Statements for Community Organizations		
	Safety	Inclusivity	Enjoyment
	Recommendations to keep children safe during activities.	Recommendations to promote a sense of belonging, celebrate diversity, reduce stigma, ensure language needs are met, and engage families as partners,	Recommendations for diverse developmentally appropriate enjoyable activities.
Structural Investment	Caregivers recommend investing in sufficient supervision to protect their children from external factors, such as outside adults, drugs, and violence. Caregivers mention various strategies to improve supervision: on-site security guards, patrolling police officers, increased recreational staff, and sufficient lighting throughout the site. Comprehensive background checks of all staff and volunteers	Requested multilingual forms and bilingual staff be available. Many families recommend culturally based activities that teach the community and their children about their cultural heritage. Caregivers of children with special needs (e.g. autism, ADHD) expressed the need for programming that can accommodate the developmental needs of their children.	Caregivers mentioned activities such as: karate, science, technology, engineering and math (STEM) activities, dance, music lessons, life skills workshops, art, and many more. Caregivers express the importance of diversity in programming to stimulate children’s different interests.
Staff and Volunteer Training	Caregivers recommend staff training on injury prevention and management, conflict resolution, and rules of the sport.	Caregivers recommend staff training on child development, supporting youth with mental and behavioral health conditions, and for children with historically marginalized communities.	Caregivers suggested offering training on positive coaching strategies.
Family-centered Approach to Activities and Engagement	A community advisory board of caregivers to alert staff about safety concerns	Caregivers recommend creating a caregiver/parent advisory board and getting input from families in the community about programming to improve service delivery. Caregivers also requested processes to facilitate communication with activity leadership about their child’s needs.	Caregivers appreciate activities where multiple family members and children of different ages can participate together, such as “Mommy and Me” classes or allowing siblings to participate together. This would help families reduce the need for childcare and create bonding opportunities for families.

### Aim 1: Motivations

#### Key Theme

Caregivers were highly motivated, believing activities facilitated lifelong healthy living and well-being for children, families, and communities. Caregivers believed activities promoted a child’s physical and socioemotional health and prepared them for future life stages. One caregiver shared, *“I hope they develop a good healthy routine… so they look for that sport or exercise as they grow up.”* They noted that activities provided space for physical activity and socioemotional skill building, such as teamwork and emotional regulation, while combating unhealthy habits like excessive screen time, substance use, and engaging in community violence. Caregivers felt youth participation improved family dynamics, reduced caregiver stress, and fostered community. One caregiver remarked, “*The after-school program works wonderfully… being a single mom*,* that helps take pressure off me.*” Activities helped caregivers build friendships, strengthen family bonds, and encourage gratitude and a desire to give back to the community.

#### Key Recommendations

More information about activities and their benefits may motivate families. One caregiver described, “*I would like more information about the activities… if we know more [we can] better motivate the parents to take their children to activities.”*

### Aim 2: Barriers: Opportunities for Access Equity

#### Key Theme

Caregivers identified barriers to youth activity participation while recognizing opportunities for equitable access. The main barriers identified included a (1) lack of awareness, (2) enrollment challenges and accessing activities, and (3) financial obstacles.

1. Lack of Awareness.

Some families lacked time or the knowledge to find suitable activities. One caregiver explained,

*Parents who are not in this area or do not have time to go to the park were unaware. Many people… are surprised at the [low] amount of money and [good] care provided to the children and they’re like*,* ‘I didn’t even know about this. And they sign up*.

(2) Enrollment and Access Challenges

Challenges included exclusive online enrollment and insufficient space for all interested children. One caregiver mentioned, “*my daughter plays softball*,* but if there’s not enough girls*,* we won’t build the team. If there’s not a coach*,* we will not build the team. I have to find another sport for her*.” Some families traveled outside their community for specialized activities like swimming, and science, technology, engineering, and math (STEM) programs.

(3) Financial Obstacles

Financial burdens, including costs related to registration, uniform, supplies, equipment, snacks, and travel expenses, deterred families. One caregiver shared, “*I had to pay [registration]*,* buy a uniform*,* and do many things*,* and I decided not to do it because I didn’t work*.”

#### Key recommendations

Parents proposed three recommendations for equitable access: (1) broad outreach using diverse strategies, (2) easy enrollment with accessible activities, and (3) low financial barriers

1. Broad Outreach Using Diverse Strategies

Caregivers recommended broad outreach using diverse strategies (e.g., emails, social media, banners, flyers, commercials) through multiple channels (e.g., parks, schools, churches) and a centralized physical and online hub for activity information across organizations.

(2) Easy Enrollment with Accessible Activities

Caregivers suggested multiple enrollment options (e.g., in-person, phone, online) with sufficient capacity for all youth to enroll in nearby activities. They recommended help lines, language-accessible low-literacy registration forms, and flexible drop-in options without prior registration required. Caregivers stressed that all children, especially those in minoritized communities, children with special needs, and girls in sports, should have equitable access to activities year-round. This includes offering diverse activities, sufficient space, and infrastructure (e.g., pools, park space) within these communities. One caregiver shared, “*I live in a predominantly Black community… A lot of us don’t know how to swim*,* and swimming can be life-saving. Every child should have the opportunity… the nearest pool is over 20 minutes away*.”

(3) Low Financial Barriers

According to caregivers, low financial barriers would increase participation. One caregiver described, “*It was $10 a week for the summer program… It was such a lifesaver*.” Caregivers recommended reducing registration costs, offering registration waivers, scholarships, installment payments, lesson-by-lesson payments, free or rental supplies/equipment, uniform assistance, providing food, and offering transportation. To offset the costs accrued by the organization, caregivers suggested that organizations find sponsors/grants, host fundraisers, partner with nonprofits, and incorporate volunteers.

### Aim 3: Definition of Quality Programs

#### Key Theme

High-quality activities consistently promote safety, inclusivity, and enjoyment. Caregivers reported varying levels of quality across their experiences with different activities, activity leaders, and organizations. Safety included protection from external (e.g., neighborhood violence) and internal (e.g., injuries) threats. Inclusivity meant fostering belonging for children from diverse backgrounds or with special needs. As one caregiver shared, “*My son has Attention Deficit/Hyperactivity Disorder (ADHD). They make sure he is focused. They don’t mind repeating things… they know his brain works differently*,* so they have him repeat it. They ensure he knows what’s going on*.” Enjoyment motivated engagement. Caregivers attributed enjoyment to the activity type, supportive and developmentally appropriate environments that built self-confidence, and opportunities to socialize and be in nature.

#### Key Recommendations

Caregivers recommended achieving consistent quality programming through (1) structural investments, (2) staff and volunteer training, and (3) family engagement.

1. Structural Investment

Caregivers recommended investing in staff, infrastructure, and programming. Safety recommendations included hiring trained staff with background clearance, installing adequate lighting, providing onsite security, and arranging escorts for families. One caregiver requested, “*Policemen will walk with them…through certain dangerous blocks*.” For inclusivity, caregivers proposed supportive programming for underrepresented youth, such as sports for girls, activities accommodating children with physical, behavioral, or developmental needs, culturally relevant activities, language accessibility, and stigma reduction initiatives, noting stigma around water sports within the Black community. Lastly, caregivers recommended funding various developmentally appropriate and enjoyable activities to encourage engagement.

(2) Staff and Volunteer Training

Caregivers recommended training for staff and volunteers. Many observed inconsistencies in instruction quality, especially among volunteers. A volunteer coach shared, “*There should be more training for volunteer coaches because they just send us out there [to]*,* figure it out*.” It was unclear whether volunteer leaders were driven by cost-cutting or community preference. While some valued volunteers to reduce costs and increase community participation, others felt it contributed to dropouts. Suggested training topics included injury prevention and management, sports rules, child development, supporting youth mental health, caring for children from historically marginalized communities, and positive coaching strategies. Caregivers hoped this training would help staff and volunteers act as supportive, caring adults.

(3) Family-centered Approach to Activities and Engagement

Caregivers requested opportunities for caregiver and sibling engagement. One caregiver proposed, *“[Have] a community advisory board. We can be an asset*,* right? Not only is our voice being heard*,* but we are helping you make this park better… so they can be part of the decision-making. For safety*,* with the board there are direct ears with the community of what’s going on*.” They also suggested processes for communication about children’s health and behavioral needs. Caregivers also appreciated options allowing siblings to participate together to simplify logistics and childcare.

## Discussion

We found that caregivers living in under-resourced communities with many Black and/or Latinx identifying residents were motivated to engage their children in activities, recognizing the potential to help children develop essential skills, enhance their physical and mental health, and contribute to family and community well-being. Caregivers proposed practical strategies to promote equitable, high-quality programming, including broad outreach with diverse methods, easy enrollment with accessible activities, low financial barriers, structural investments, staff and volunteer training, and family engagement. Addressing structural causes of access and quality inequities may boost engagement and health equity.

Our findings align with the Health Equity Framework and build on earlier surveys. Caregivers recognized the impact of structural disinvestment as a key obstacle (*Systems of Power Domain*), linking disparities to missing infrastructure, such as pools and insufficient park space. Consistent with other studies, caregivers also highlighted cost as a primary barrier (Afterschool Alliance, 2021; Kelly Hinchcliffe, 2023). These interviews described other prohibitive costs like equipment and transportation. New barriers also emerged, including outreach and enrollment. Within the *Individual Factor*,* Physiological Pathways*,* and Relationships and Networks Domains*, caregivers noted skill development and physical health benefits, aligning with prior survey findings (Afterschool Alliance, 2021; Kelly Hinchcliffe, 2023). These interviews identified underexplored benefits, including mental health and family well-being, which are particularly relevant given the rise in youth and adult mental health challenges and is consistent with quantitative studies (La Charite et al., 2023; Parodi et al., 2022; Terlizzi & Zablotsky, Benjamin, 2024; Wilson & Dumornay, 2022).

Our study amplifies caregiver voices in under-resourced communities, offering their recommendations for improving access and quality equity in youth activities. Similarities to national surveys suggest broader applicability of our qualitative findings, while the narratives refine prior survey results (Afterschool Alliance, 2021; Kelly Hinchcliffe, 2023). Our findings can inform a caregiver-centered framework and survey tools for assessing ongoing activity access and quality, which was supported by the theme requesting family engagement. Researchers can evaluate whether caregiver-informed interventions boost engagement in health-promoting activities.

There are limitations to consider. Participants were recruited from one urban county in the United States, and eligibility was restricted to caregivers proficient in English or Spanish. The findings may be transferable to similar urban counties with a history of disinvestment and significant proportions of low-income Black and Latinx residents. We could not achieve cultural congruence for the participants who identified as Black or African American. Although we aimed to reach families that were and were not involved in activities, most families had current or past involvement, consistent with national data (La Charite et al., 2023).

The findings have implications for professionals and organizations who work with youth (e.g., activity providers, school nurses or counselors, pediatric clinicians). To facilitate child enrollment, youth-facing professionals can discuss activities as health-promoting tools, provide strategies to overcome barriers, and maintain up-to-date lists of low-barrier, high-quality activities in their area. Pediatric organizations may consider developing guidelines to inform training materials to ensure activity leaders are equipped to support children with special health needs.

Public agencies and activity organizations can collaborate to build a unified activity system, integrating tools for monitoring and continuous improvement to achieve equitable access and quality. Existing efforts include intermediary organizations aiding smaller organizations in offering activities and frameworks like Every Hour Counts for Measurement, Continuous Improvement, and Equitable Systems (Every Hour Counts, 2021). Our study emphasizes prioritizing family voices in these system-level improvements.

Policymakers and child health advocates should prioritize investments in infrastructure (parks, pools, lighting), reducing family costs, staff and volunteer training, and ensuring equitable access to specialized activities (e.g., swimming, STEM). This is important as the Coronavirus-2019 era emergency relief funding for afterschool programs ends. One caregiver noted, “*It is important to build community pride in our children. If these services are not offered*,* that sense of community will be lost; we need to invest in them*.”

## Conclusion

Caregivers in under-resourced communities with high proportions of residents who identify as Black and/or Latinx are highly motivated to engage their children in out-of-school activities for skill development, relationship building, and overall well-being. They offer practical suggestions to enhance accessibility, quality, and equity in youth activities. Future efforts can assess whether these themes are consistent among different subpopulations or geographic regions and investigate whether increased caregiver input helps reduce disparities in activity participation.

## Data Availability

Interview recordings are not available for participant protection.

## Appendix

See Tables 5, 6 and 7.

**Table 5:** Aim 1: additional caregiver motivation quotes

Aim 1: Motivations		
Key Theme	Key Theme Elements	Quotes
Caregivers were highly motivated, believing that activities were facilitators of lifelong healthy living and wellbeing for children, families and communities	Healthy Habits	“Mi hijo siempre ha tenido sobrepeso… Aprenden a hacer etiquetas en alguna de las clases que van… pero ya tienen la conciencia de que algo no está bien, que tiene demasiada sal o demasiada azúcar. Me gusta escucharlos. Empiezan una conversación entre ellos como hermanos.” *“My son has always been overweight… They learn to read labels and in one of the classes they go to. They are aware that… there is too much salt or too much sugar. I like to listen to them. They start a conversation with each other and like brothers.”*
	Skill Development	“Being part of sport requires a lot of… it’s a regimen. There’s a routine, there’s a schedule so I feel it helps in everything as they get older. They get familiar with employment.”
	Deter Unhealthy Habits	“I wanted him to get off that PlayStation. He’s always on that PlayStation. And get him off all those tablets.”
	Physical Health	“Sí porque hacen un poquito de ejercicio y es algo diferente a que estén en la casa, yo vivo en un apartamento y no tengo mucho espacio entonces ayuda mucho a su desarrollo físico o emocional.” *“They get a little exercise*,* and it is something different from being at home. I live in an apartment*,* and I don’t have much space*,* [it] helps a lot with their physical or emotional development.”*
	Mental Health	“These children have a place to go to have fun and be free… something to look forward to, its happiness, its joy. It helps tremendously with their mental health – they have a happy environment to thrive.”
	Family Wellbeing	“Estos recreativos sí nos ha ayudado mucho a tener la familia más unida y todo… Pasamos más tiempo juntos. Eso nos ha ayudado.” *“These recreational activities have helped us to have a more united family and everything… We spend more time together. That has helped us.”*
	Community Wellbeing	“Maybe in the future… as a grateful person, he will become a coach for the community… to give back to the community.”
Recommendations	Information about Activity	See in-text quote

**Table 6:** Aim 2: additional caregiver quotes on barriers and improving access

Aim 2: Barriers & Improving Access		
Key Theme	Key Theme Elements	Quotes
Caregivers identified ongoing barriers to youth activity participation while also recognizing opportunities to improve equitable access to activities	Awareness	See in-text quote
	Outreach	“It gets confusing when you make the [online] account -- it’s so many activities that you get lost -- the pool has its own website… it has swimming lessons, private lessons, free time, ‘Oh, I don’t know what to do.’ I prefer to be safe by just going to the physical park, because I know that I can communicate with someone. I don’t know if a helpline would help. I’m pretty tech-savvy, but get confused with the website.”
	Accessibility	“Los parques sí quedaron un poquito lejos… a veces están un poquito como descuidados y a veces cuando-, sólo los fines de semana están bien llenos.” *“The parks were far away… they are a bit neglected and sometimes only the weekends they are very full.”*
	Financial	“Se juntaban en diferentes parques… yo no tenía carro, entonces andar en el bus con ellos, gastar más dinero… hay gente que no tiene trabajo y hasta 5 dólares cuando no hay es demasiado” *[They] met in different parks… I didn’t have a car*,* so riding the bus*,* spending even more money… there are people who don’t have a job and even $5 when there isn’t any is too much.*

Recommendations	Subthemes	Quotes
	Awareness/Outreach	Estaría mucho mejor que mandaran más folletos a las escuelas para que tuviera una mayor información. *“It would be much better if they sent more brochures to schools so they’d have more information.”*
	Enrollment	“I prefer… going to the physical park because I know I can communicate with someone. I don’t know if a helpline would help. I’m pretty tech-savvy but get confused with the website.”
	Accessibility	See in-text quote
	Financial	“I think it was $10 a week for the summer program, which I could have cried. It was such a lifesaver; I couldn’t afford for my two children to go to a summer program that is as pricey as normal summer programs.”

**Table 7:** Aim 3: caregiver quotes on quality definition and recommendations

	Key Theme: Caregivers described high-quality activities as those that consistently promoted safety, inclusivity, and enjoyment.		
	Safety	Inclusivity	Enjoyment
Key Theme	“The ratio of adult supervision to students is really good. So it makes me feel more comfortable, because I feel like my children are being monitored, because kids get into a lot nowadays.”	“Estaba un niño hace dos años, el niño llegó-, llegó en silla de ruedas aquí en la School, y él dijo “Quiero hacer Cheerleader” Personas que tuvieron ahí, trabajaron muy bien con él y lo hicieron que incluyera, participará y hacía todos los bailes, los movimientos y él lo hizo muy bien, me gustó mucho.” *“There was a boy two years ago, the boy came-, he arrived in a wheelchair here at the School, and he said "I want to be a Cheerleader." I didn't know anything, but those who were-, people who were there, worked very well with him and they made him feel included, he participated and he did all the dances, the movements and he did it very well, I liked it a lot.”*	“Usually he would be quitting sport right now but he loves it.”
Structural Investment	“Pues que haya más seguridad en los parques… si un parque está lleno de niños y de padres, las personas sin hogar o las personas que hacen-, drogas, no- no van a estar ahí ¿Verdad?… Lo que más priorizó es la seguridad.” *There should be more security in the parks… if a park is full of homeless people or people who do drugs*,* [families] are not going to be there*,* right?… What I prioritize the most is security.*	“Como poner más sobre culturas, porque somos diferentes, de diferentes países venimos… por lo menos, podemos educar a los niños, a los- a la comunidad sobre nuestras raíces que traemos, todos, diferentes, para poder este-, no se pierda, pues, la igualdad para todos. *Put more about cultures, because we are different, we come from different countries… so that at least we can educate the children, the- the community about our roots that we bring, all of us, different*	“Quizás más opciones para los niños, que escojan y no estén tan limitada las actividades.” “*Perhaps re more options for children to choose from and not be so limited in activities”*
Staff and Volunteer Training	“In terms of safety, you want to [pay] attention to detail with interaction between kids behavior. Thinking outside the box, thinking of ways you can build camaraderie, bring folks together, not create friction or division and you don’t want to fan the flames if there is hostility amongst the children and stuff.”	“When there’s something going on, they call the parent to come and pick him up. There are no sit downs, no trying to see if the kid is okay at home. They don’t see if the child is okay mentally, emotionally.”	“Me gustaría también que hubiera más capacitación en cuanto a… como motivar a los niños… y que no digan cosas que puedan ser contraproducentes y entonces en lugar de motivarlos los-, los orillan a dejar de hacer las cosas que les gustan” *I would like there to be more training…in terms of; how to motivate the children and not say things that could be counterproductive and then instead of motivating them*,* they force them to stop doing the things they like.*

## References

[CR1] Afterschool Alliance (2021). *America after 3pm for Black families and communities.* https://afterschoolalliance.org/documents/AA3PM-2020/AA3PM-Black-Communities-2020-Fact-Sheet.pdf

[CR2] Afterschool Alliance (2022). *Access to Afterschool Programs Remains a Challenge for Many Families*. 1–5.

[CR3] America, & Becoming (2001). : *Racial Trends and Their Consequences, Volume 1* (p. 9599). National Academies Press. 10.17226/9599

[CR5] Braun, V., & Clarke, V. (2006). Using thematic analysis in psychology. *Qualitative Research in Psychology*, *3*(2), 77–101. 10.1191/1478088706qp063oa

[CR7] County of Los Angeles, California State Parks (2024). *Children with Easy Access to a Safe Place to Play*. https://data.lacounty.gov/datasets/lacounty::children-with-easy-access-to-a-safe-place-to-play/explore?filters=eyJQbGF5X1BsYWNlIjpbNDguNiw5N119

[CR8] DeCamp, L. R., Showell, N., Godage, S. K., Leifheit, K. M., Valenzuela-Araujo, D., Shah, H., & Polk, S. (2019). Parent activation and pediatric primary care outcomes for vulnerable children: A mixed methods study. *Patient Education and Counseling*, *102*(12), 2254–2262. 10.1016/j.pec.2019.07.004 31288957 10.1016/j.pec.2019.07.004 PMC7266441

[CR9] *Dedoose* (2024). https://www.dedoose.com/?gad_source=1&gclid=CjwKCAiAmfq6BhAsEiwAX1jsZ7HuBpBgLWA9VlnIleyk5TA3BhpJ_nEkgrAJCsqlJePCLEJIRgt_ARoCBKAQAvD_BwE.

[CR10] Donnelly, M., Lažetić, P., Sandoval-Hernandez, A., Kameshwara, K., & Whewall, S. (2019). An Unequal Playing Field: Extra-Curricular Activities, Soft Skills and Social Mobility. Social Mobility Commission. https://www.researchgate.net/publication/346954878_An_Unequal_Playing_Field_Extra-Curricular_Activities_Soft_Skills_and_Social_Mobility

[CR11] Duerden, M. D., Witt, P. A., & Harrist, C. J. (2013). The impact of parental involvement on a structured youth program experience: A qualitative inquiry. *Journal of Youth Development*, *8*(3). 10.5195/jyd.2013.88

[CR12] Easterlin, M. C., Chung, P. J., Leng, M., & Dudovitz, R. (2019). Association of team sports participation with Long-term mental health outcomes among individuals exposed to adverse childhood experiences. *JAMA Pediatrics*, *173*(7), 681–688. 10.1001/jamapediatrics.2019.1212 31135890 10.1001/jamapediatrics.2019.1212 PMC6547068

[CR13] ETR (2024). *ETR’s Health Equity Framework*. Advacing Health Equity. https://www.etr.org/about-us/health-equity-framework/

[CR14] Every Hour Counts. (2021). Putting data to work for young people: A framework for measurement, continuous improvement, and equitable systems. *Wallace Foundation*, 21. 10.59656/YD-OS7384.001

[CR15] Fisher, K. M., Shannon-Baker, P., Greer, K., & Serianni, B. (2022). Perspectives of students with disabilities and their parents on influences and barriers to joining and staying in extracurricular STEM activities. *The Journal of Special Education*, *56*(2), 110–120. 10.1177/00224669211054109

[CR16] Frey, W. (2021). *2020 Census: Big cities grew and became more diverse, especially among their youth*. Brookings. https://www.brookings.edu/articles/2020-census-big-cities-grew-and-became-more-diverse-especially-among-their-youth/

[CR17] Galperin, L. A. C. R. (2021). *A Great Divide: L.A. Equity Index*. ArcGIS StoryMaps. https://storymaps.arcgis.com/stories/ca477e68657643c9a2bad1fddfe24359

[CR18] Gleeson, M., Bishop, N. C., Stensel, D. J., Lindley, M. R., Mastana, S. S., & Nimmo, M. A. (2011). The anti-inflammatory effects of exercise: Mechanisms and implications for the prevention and treatment of disease. *Nature Reviews Immunology*, *11*(9), 607–615. 10.1038/nri3041 21818123 10.1038/nri3041

[CR19] Guilmette, M., Mulvihill, K., Villemaire-Krajden, R., & Barker, E. T. (2019). *Past and present participation in extracurricular activities is associated with adaptive self-regulation of goals, academic success, and emotional wellbeing among university students*. 10.1016/j.lindif.2019.04.006

[CR20] Heath, R. D., Anderson, C., Turner, A. C., & Payne, C. M. (2022). Extracurricular activities and disadvantaged youth: A Complicated—But Promising—Story. *Urban Education*, *57*(8), 1415–1449. 10.1177/0042085918805797

[CR21] Jones, L., & Wells, K. (2007). Strategies for academic and clinician engagement in Community-Participatory partnered research. *Journal of the American Medical Association*, *297*(4), 407. 10.1001/jama.297.4.407 17244838 10.1001/jama.297.4.407

[CR22] Ju, B., Ravenscroft, O., Flores, E., Nacu, D., Erete, S., & Pinkard, N. (2020). Understanding Parents’ Perceived Barriers to Engaging Their Children in Out-of-School STEM Programs. *2020 Research on Equity and Sustained Participation in Engineering, Computing, and Technology (RESPECT)*, *1*, 1–4. 10.1109/RESPECT49803.2020.9272451

[CR23] Kallio, H., Pietilä, A. M., Johnson, M., & Kangasniemi, M. (2016). Systematic methodological review: Developing a framework for a qualitative semi-structured interview guide. *Journal of Advanced Nursing*, *72*(12), 2954–2965. 10.1111/jan.13031 27221824 10.1111/jan.13031

[CR24] Kelly Hinchcliffe (2023). *After-School Program Trends Reporters Should Cover*. Education Writers Association. https://ewa.org/how-to-cover-the-story/barriers-to-kids-accessing-after-school-programs

[CR25] Kephart, L. (2022). How Racial residential segregation structures access and exposure to greenness and green space: A review. *Environmental Justice*, *15*(4). 10.1089/env.2021.0039

[CR26] Klein, R. J. (2001). Los Angeles County Population Estimates. *National Center for Health Statistics*. http://www.lapublichealth.org/epi/docs/2017-LAC-Population.pdf

[CR27] La Charite, J., Macinko, J., Hedrick, R., Santoro, M., & Dudovitz, R. (2023). Extracurricular activities, child and caregiver mental health, and parental Aggravation-A National Cross-Sectional study. *Academic Pediatrics*, *23*(7), 1394–1402. 10.1016/j.acap.2023.01.001 36634843 10.1016/j.acap.2023.01.001

[CR28] Lincoln, Y. S., & Guba, E. G. (1985). *Naturalistic inquiry*. Sage.

[CR29] Los Angeles County Department of Public Health (2014). *Supplement to Community Health Assessment Service Planning Area 7: East*. http://publichealth.lacounty.gov/plan/docs/SPA7Supplement.pdf

[CR30] Mahoney, J. L., Schweder, A. E., & Stattin, H. (2002). Structured after-school activities as a moderator of depressed mood for adolescents with detached relations to their parents. *Journal of Community Psychology*, *30*(1), 69–86. 10.1002/jcop.1051

[CR31] McKenzie, T. L., Moody, J. S., Carlson, J. A., Lopez, N. V., & Elder, J. P. (2014). *Neighborhood Income Matters: Disparities in Community Recreation Facilities, Amenities, and Programs*. PMC4082954 25006598

[CR32] Naff, D., Hale, R., Simmons, J., Gaston, A., Wright, L. Y., Bradford, K., Bell, C. R., Flynn, J., Creasy, C., Guerrero, Y., Dunn, R., & Weaver, P. (2024). *Understanding the role of out of school time (OST) providers in urban school systems in a Post-COVID context*. Metropolitan Educational Research Consortium Institute for Collaborative Research and Evaluation. https://scholarscompass.vcu.edu/cgi/viewcontent.cgi?article=1145&context=merc_pubs

[CR33] National Academies of Sciences, Engineering, and Medicine (2019). *Shaping Summertime Experiences: Opportunities to Promote Healthy Development and Well-Being for Children and Youth* (M.-J. Sepúlveda & R. Hutton, Eds.; p. 25546). National Academies Press. 10.17226/25546 31940162

[CR34] Oberle, E., Ji, X. R., Kerai, S., Guhn, M., Schonert-Reichl, K. A., & Gadermann, A. M. (2020). Screen time and extracurricular activities as risk and protective factors for mental health in adolescence: A population-level study. *Preventive Medicine*, *141*. 10.1016/J.YPMED.2020.106291 10.1016/j.ypmed.2020.106291 33069689

[CR35] Ong, P., & Firestine (2008). Theresa, pfeiffer, deirdre, poon, Oiyan. In L. Tran (Ed.), *The state of South LA*. UCLA School. https://knowledge.luskin.ucla.edu/wp-content/uploads/2018/01/The_State_of_South_LA.pdf of Public Affairs.

[CR36] Parodi, K. B., Holt, M. K., Green, J. G., Porche, M. V., Koenig, B., & Xuan, Z. (2022). Time trends and disparities in anxiety among adolescents, 2012–2018. *Social Psychiatry and Psychiatric Epidemiology*, *57*(1), 127–137. 10.1007/s00127-021-02122-9 34100110 10.1007/s00127-021-02122-9 PMC8183580

[CR37] Phillips, W., Dufresne, Eliane, Larmour, S., & Speciani, E. R. (2021). Benefits of extracurricular activities for children. *Publications Office of the European Union*. https://op.europa.eu/en/publication-detail/-/publication/c2c8d076-0a04-11ec-b5d3-01aa75ed71a1/language-en

[CR38] Schwartz, G. L., Wang, G., Kershaw, K. N., McGowan, C., Kim, M. H., & Hamad, R. (2022). The long shadow of residential Racial segregation: Associations between childhood residential segregation trajectories and young adult health among black US Americans. *Health & Place*, *77*, 102904. 10.1016/j.healthplace.2022.102904 36063651 10.1016/j.healthplace.2022.102904 PMC10166594

[CR4] A.Smith, J., Harr&, R., #233, VanLangenhove, L., & Smith, J. A. (1995). Semi-Structured Interviewing and Qualitative Analysis. In *Rethinking Methods in Psychology* (pp. 10–26). SAGE Publications Ltd. 10.4135/9781446221792.

[CR39] Terlizzi, E., & Zablotsky, Benjamin, B. (2024). *Symptoms of anxiety and depression among adults: United states, 2019 and 2022*. National Center for Health Statistics (U.S. 10.15620/cdc/164018 10.15620/cdc/64018 PMC11616099 39591466

[CR40] The Child and Adolescent Health Measurement Initiative (CAHMI) (2025). *NSCH Survey Instruments*. Data Resource Center for Child and Adolescent Health. https://www.childhealthdata.org/learn-about-the-nsch/survey-instruments

[CR6] Tong, A., Sainsbury, P., & Craig, J. (2007). Consolidated criteria for reporting qualitative research (COREQ): A 32-item checklist for interviews and focus groups. *International Journal for Qualitative in Health Care, 19*(6), 349–357. 10.1093/intqhc/mzm042 10.1093/intqhc/mzm042 17872937

[CR41] Turale, S. (2020). A brief introduction to qualitative description: A research design worth using. *PRIJNR*, *24*(3). https://he02.tci-thaijo.org/index.php/PRIJNR/article/view/243180

[CR42] Wilson, S., & Dumornay, N. M. (2022). Rising rates of adolescent depression in the united states: Challenges and opportunities in the 2020s. *The Journal of Adolescent Health: Official Publication of the Society for Adolescent Medicine*, *70*(3), 354–355. 10.1016/j.jadohealth.2021.12.003 35183317 10.1016/j.jadohealth.2021.12.003 PMC8868033

